# Supplementary material for: Uncertainty-aware breeding decisions: MCMC-based optimum contribution selection increases breeding decision robustness
Source: Genetics. 2026 Aug 3;234(2):iyag205. doi: 10.1093/genetics/iyag205 (PMC13643769; doi:10.1093/genetics/iyag205)
Supplement: Supplementary file 1 [file iyag205_peer_review_history.pdf]

GENETICS

Uncertainty-aware breeding decisions: MCMC-based optimum contribution selection increases breeding decision robustness

Jon Ahlinder and Patrik Waldmann

NOTE: The reviews and decision letters are unedited and appear as submitted by the reviewers.
In extremely rare instances and as determined by a Senior Editor or the EIC, portions of a review may be redacted. If a review is signed, the reviewer has agreed to no longer remain anonymous.
The review history appears in chronological order.

Review Timeline:

Submission Date:	2026-03-12
Editorial Decision:	2026-04-19
Resubmission Received:	2026-06-04
Editorial Decision:	2026-06-30
Revision Received:	2026-07-14
Accepted:	2026-07-19

April 19, 2026

GENETICS-2026-309187
Uncertainty-aware breeding decisions: MCMC-based optimum contribution selection increases breeding decision robustness

Dear Dr. Ahlinder:

Two experts in the field have reviewed your manuscript, and I have read it as well. The reviewers recognized the significance of the research idea, as well as the quality of the writing and implementation. While your manuscript is not currently acceptable for publication in GENETICS, we would welcome a substantially revised manuscript that addresses the reviewer comments. You can read their reviews at the end of this email.

All reviewer comments must be addressed in your point-by-point response. In particular, your hypothesis that the MCMC-OCS method improves long-term selection compared to MAP-OCS has not been critically tested; nor has the practical significance of the robustness scores. Simulation results are needed to show the impact of these new methods.

We look forward to receiving your revised manuscript. Please let the editorial office know approximately how long you expect to need for revisions.

Upon resubmission, please include:
1. A clean version of your manuscript;
2. A marked version of your manuscript in which you highlight significant revisions carried out in response to the major points raised by the editor/reviewers (track changes is acceptable if preferred);
3. A detailed response to the editor's/reviewers' feedback and to the concerns listed above. Please reference line numbers in this response to aid the editor and reviewers.

Your paper will likely be sent back out for review.

Additionally, please ensure that your resubmission is formatted for GENETICS
https://academic.oup.com/genetics/pages/general-instructions

Follow this link to submit the revised manuscript: Link Not Available

Sincerely,

Jeffrey Endelman
Associate Editor
GENETICS

Approved by:
Hongyu Zhao
Senior Editor
GENETICS

Reviewer #1 :

This is a very well-written manuscript that has the potential to advance the field of optimising selection decisions in breeding programs. Particularly as the method appears to be, to some extent, a formalisation and extension of the method proposed by Pocrnic et al. Furthermore, the comparison metrics used are interesting and useful, particularly for interpreting uncertainty effects. Unfortunately, it lacks clarity on critical information and discussion to make the proposed methodology appealing to other breeding programs (think about generalisation across species!) beyond those discussed by the authors. In particular, there is a lack of clarity regarding computational scalability, the methodology used on the tree data, and the potential for routine implementation.

Major/General comments:
The tested data is quite limited for the proper assessment and generalisation. Due to the long generation interval on trees, it is impossible to use the real data to properly assess the outcomes of the proposed methods to future gain and diversity, for example, by masking data from the last generation and evaluating how resulting selection candidates (and their GEBV, ...) compare to the realised ones (or some other measures of forward validation that would compare realised genetic trend by

potential trend using the novel OCS method).

Furthermore, in the methods section, it is not clear which model was used, with how many traits, with how many phenotypic datapoints, and how many individuals in pedigree (A) and with genotypes (G) were available in total in the analysis (particularly for Norway Spruce). Also, why was the H matrix used for Norway spruce and not for Loblolly? For H, you report Tau and Omega, why would that be important in your case and why don't you report the alpha and beta values?

Why was there a need to use a complex Bayesian mixture model (correlated continuous + categorical traits) for testing purposes? Is this really the standard model applied in the breeding programmes?
How do your genetic constraints of 0.02 and 0.03, transform into standardly used constrain on rate of inbreeding per generation. And, how do the results affect or depend on the group coancestry constraint (Θ)?

When we entered the genomic era, there was a big question: which matrix should be used in the OCS step (A, G, H, ???), and which in the breeding value estimation step? Have you tested the sensitivity of your method, and/or could you provide some discussion on this? I mean it is clear you did A,G, and H on Norway data.

There is a lack of generalisation needed for the methodology to be assessed. Given that testing on a different species (especially in nucleus breeding programs like chicken or pig populations that would benefit the most) is not feasible (I assume?), I recommend using a small, simulated example to assess and compare the long-term outcomes of the proposed methodology with those of the standard selection and optimisation methods. Also, it is difficult to determine if the method really accounts for uncertainty, as it is assessed only using GEBV rather than true breeding values. A validation strategy within simulation incorporating TBV would show this!

Furthermore, I am worried about the extremely low number of MCMC samples. The authors use just 1000, whereas in real livestock datasets, the orders are in the 100,000s. So, the biggest drawback is the lack of a proper assessment of the computational complexity of that method. How does the method scale computationally for larger datasets? In fast-turnaround breeding programs, even regular OCS is avoided due to these computational complexities. Thus, the authors need to include computational aspects and computational scalability in the manuscript. You can start by taking parts from the supplement and add to the main text!

Furthermore, in the MCMC, increasing the number of samples should be accompanied by convergence diagnostics that are not clearly reported. This is essential for validating the approach.

Minor comments:

Page 2, Line 35: The term "underestimate" may not always be appropriate. In some cases, it could be an overestimate, or even equal. This should be clarified.
Page 2, Lines 56-58:The statement that "ignoring uncertainty of EBV leads to suboptimal decisions" needs refinement. EBVs are, by definition, unbiased estimators of breeding values. The issue is not the EBV itself but rather ignoring uncertainty in the selection process. This distinction should be made clearer.
Page 4, Line 83: Reference to Section 2.6 appears incorrect.
Page 5, Line 29: Reference to Section 2.5 appears incorrect - Please check internal cross-referencing throughout the manuscript.
Figure 2c: It seems to be mentioned but not explicitly referenced in the text.
Table 4 (Overlap metric): It is unclear whether overlap is reported as a count or a percentage. The manuscript is inconsistent: Some sections report percentages, others report counts. If it is a count, values such as 26.4 individuals are not meaningful (should be integer values). Similarly, 17.5 individuals (Page 6, Line 71) are confusing - Ensure consistency and clarity in how this metric is defined and reported.
Interpretation of overlap count metric: The classification of low, medium, and high overlap is unclear and somewhat subjective. For example: 26.6% is described as high and 17.5 as moderate - reducing uncertainty analysis to this metric without clear thresholds or justification may be misleading.
The data availability statement includes only Norway spruce; please add a statement on other datasets and on general code availability.

Reviewer #2 :

This paper proposes a new idea of studying the impact of posterior uncertainty in EBVs (estimated breeding values) on OCS (optimum contribution selection). The authors obtain posterior EBV samples from a Bayesian mixed model, rerun OCS across MCMC draws, compare the results against the point-estimate MAP-OCS method, and propose several metrics to evaluate the robustness and contribution. The authors conduct extensive analysis on two real-data studies in Norway spruce and Loblolly pine and show that posterior uncertainty can substantially change which individuals are selected within families.

There are several concerns that I would like to share

1. Equation 8 introduces a generic random effect a, but equation 9 switches to g1 and g2 without explicitly linking them. The covariance notation in L25-26 and L37 is partly inconsistency, is the joint covariance Cov(y) dimensionally complete without the relationship matrix G? The residual term changes the notation from R to R0 which causes confusion. In L36, should the correlation formula be defined as covariance divided by square root of product of variances?
2. The Bayesian setup is underreported. It is mentioned that the authors used JWAS with "default settings" and 1000 MCMC iterations, but they do not show the priors for fixed effects, variance components, etc. which should be worth mentioning since they are key modeling structure for the readers to understand.
3. The Spearman correlation between c_{MAP} and c_j is less convincing. Many individuals may have close to zero contributions in OCS and rank correlation over individuals can be dominated by ties and sparse structure. This metric may not explain much more than the overlap metrics as Table 4 already shows these correlations are near zero in all datasets.
4. The robustness score is actually a leave-one-out replaceability score since it is defined as the loss in expected gain when an individual is removed and OCS is rerun and being averaged over MCMC iterations. The reason of having a low score can be the case that one candidate has many close substitutes, not because of uncertain EBV.
5. Low overlap between MAP-OCS and MCMC-OCS is very interesting to observe, but this does not automatically prove that MCMC-OCS gives better results. We may need simulation with known truth or external validations to prove this.

Reviewer #3 :

The paper explored the effects of uncertainty of point mass estimation in Optimal Contribution selection, using two distinct datasets to test the implementation. The authors develop an MCMC-based robustness-score metric that quantifies the effects of breeding-value uncertainty on individual importance. While it is a relevant, high-impact topic in breeding, it remains poorly documented, underscoring the importance of the paper.

The authors should add some small changes as indicated below:

1. The robustness score threshold is arbitrary and underexplored. The authors explicitly acknowledge that the bottom 25% cutoff for "high-risk" classification is arbitrary. However, I would add some more intuition/explanation to guide a practical decision by someone implementing this tool in the discussion section.
2. The paper uses G (genomic, IBS-based) and A (pedigree, IBD-based) matrices somewhat interchangeably in the OCS constraint, but the theoretical properties of OCS assume IBD-based kinship. The authors show that using A vs G changes overlap metrics (Table 4), but they do not discuss what this means for the interpretation, once the parametric space of A/G are different and the same threshold was used for coancestry (0.02 and 0.03 for the two datasets).
3. The increase in Gini coefficient and top-10% concentration in Norway spruce after excluding high-risk individuals is mechanistically explicable; constrained re-optimization with a reduced candidate pool will naturally concentrate contributions among remaining elite individuals. However, this result exposes a conceptual tension in the dual-metrics framework: individual robustness and portfolio concentration do not necessarily move together, and the paper provides no principled guidance on how to weight these competing dimensions of robustness when they conflict.
The contrasting behavior between Norway spruce and Loblolly pine, where metrics were concordant in the latter but divergent in the former (likely reflects differences in coancestry constraint tightness and candidate pool size), but this mechanistic explanation is absent from the discussion.
4. Figure 3b shows some high-contribution individuals flagged as high-risk; this is one compelling result in the paper and deserves more discussion.
5. Page 5, line 80: It seems that the constraint mentioned here is in relation to the Loblolly pine dataset. However, the authors already mentioned this in lines 62-63 (Page 3).
6. Page 13, line 67: "Here, we have also provided a practical decision-making framework with robustness metrics and replacement strategies which can be utilized to boost long-term stability of breeding programs." The authors claimed that they presented a practical decision-making framework; however, it was based on the 25% threshold, which was arbitrary, where you mentioned: "we are interested in showing the principle rather than giving an absolute recommendation" (Page 4, 75-80). So, I would just tune down a little on this sentence, once the 25% threshold would be debatable, depending on the breeding program.
7. Page 4, line 83: (Section 2.6) could not find this section.

Associate Editor Comments:

P2 L29 "robust OCS preferred individuals with low SEs even if they had high EBV". In this example, were lower trait values preferred? The default would be to assume that high EBV and low SE is the preferred combination.

P4 L93 I would encourage you to select a different name than "contribution" for the capital C in Eq. 25 because contribution is the accepted name for the lower case c.

The reference for Fogg et al. (2024) needs an arXiv identifier or URL.

The reference for Pocrnic et al. (2023) is incomplete as the book name is missing.

Response to Reviewers

GENETICS-2026-309187

Uncertainty-aware breeding decisions: MCMC-based optimum contribution selection increases breeding decision robustness

Jon Ahlinder and Patrik Waldmann

27 May 2026

Associate Editor (Prof. Jeffrey Endelman)

> *P2 L29: "robust OCS preferred individuals with low SEs even if they had high EBV". In this example, were lower trait values preferred? The default would be to assume that high EBV and low SE is the preferred combination.*

Response:
Thank you for this clarifying question. The sentence was ambiguous. The original reference is to Fogg et al. (2024), whose robust OCS penalizes candidates whose high EBVs are accompanied by high standard errors (large uncertainty), so that — under strong risk aversion — an individual with a moderately high but precisely estimated EBV can be preferred over an individual with a higher but less reliable EBV estimate. The intended meaning is not that low trait values are preferred, but that reliability can outweigh raw magnitude in the robust framework. We have reworded this sentence in the revised Introduction to make the mechanism explicit:

> *"The robust OCS penalizes candidates whose high EBVs are accompanied by large SEs, preferring individuals with more reliable estimates even at some cost to expected gain; as the risk-aversion parameter approaches its maximum, this framework reduces to a minimax worst-case formulation that guards against the single most adverse realization of EBV uncertainty."*

> *P4 L93: I would encourage you to select a different name than "contribution" for the capital C in Eq. 25 because contribution is the accepted name for the lower case c.*

Response:
Agreed. The quantity previously labelled C_i (the product of optimal contribution c_i and breeding value BV_i, representing the expected genetic gain attributable to individual i) has been renamed throughout to the *marginal gain* of individual i, denoted M_i. All equations, table headers, and figure captions in the supplementary material have been updated accordingly.

> *The reference for Fogg et al. (2024) needs an arXiv identifier or URL. The reference for Pocrnic et al. (2023) is incomplete as the book name is missing.*

Response:
Both references have been corrected in the bibliography. Fogg et al. (2024) now includes the arXiv identifier [please confirm current arXiv ID before final submission]. The Pocrnic et al. (2023) entry now includes the full book title (*Proceedings of the 12th World Congress on Genetics Applied to Livestock Production*).

Reviewer 1

We thank Reviewer 1 for their thorough and constructive assessment. We address the major and minor comments in order.

Major/General Comments

> *The tested data is quite limited for the proper assessment and generalisation. [...] I recommend using a small, simulated example to assess and compare the long-term outcomes of the proposed methodology with those of the standard selection and optimisation methods. [...] A validation strategy within simulation incorporating TBV would show this!*

Response:
This is the central concern, and we have addressed it fully. We added a complete simulation study using the QTL-MAS 2010 workshop dataset (Szydlowski et al. 2011), which provides a multi-generation genomic selection scenario (n = 3,226 individuals, five generations, two correlated traits, p = 9,723 SNPs) with known true breeding values for all individuals. The n = 900 fifth-generation candidates served as the selection pool; their GEBVs and full posterior distributions were estimated with a bivariate Bayesian GBLUP model in JWAS (I = 5,000 post-burn-in MCMC samples).

An oracle solution MAP-OCS(TBV) — computed from true rather than estimated breeding values — served as the theoretical benchmark. Key findings are:

- MAP-OCS overestimated its own expected genetic gain by 16.7% relative to its realised in-sample gain across MCMC scenarios, a bias arising from treating posterior-mean EBVs as fixed truth. CVaR-OCS is free of this bias by construction.
- At the recommended operating point (α = 0.95, μ = 1.50, Θ = 0.03), CVaR-OCS improved CVaR95 by +1.26% at negligible gain cost (−0.04%).
- CVaR-OCS recovered one additional oracle-optimal individual (11 vs. 9 shared with oracle; Jaccard +11%) and reduced gain-distribution variance by 4.8%, without access to true breeding values.

These results appear in a new subsection *Validation on a simulated dataset with known true breeding values*, with Table 2 and Figure 5 in the main text. We acknowledge that a single-generation benchmark cannot fully characterise long-term trajectories, and this limitation is explicitly stated in the Discussion and Abstract.

> *In the methods section, it is not clear which model was used, with how many traits, with how many phenotypic datapoints, and how many individuals in pedigree (A) and with genotypes (G) were available in total (particularly for Norway Spruce). Also, why was the H matrix used for Norway spruce and not for Loblolly? For H, you report Tau and Omega, why would that be important in your case and why don't you report the alpha and beta values?*

Response:
Thank you; these details were insufficiently documented. We have added a structured Table 1 (Description of included datasets) summarising pedigree size, candidate pool, generations, number of families, average coancestry, and propagation mode for all datasets. The Norway

spruce Methods subsection now explicitly states: 3 traits for the G-matrix model (n = 1,218; Hjd17, Htv17, Sprant17) and 4 traits for the H-matrix model (n = 5,525; adding Lev17 as a threshold trait); phenotypic record counts and missingness are in the new Supplementary Table S1.

Regarding why the H matrix was used for Norway spruce but not Loblolly: the Norway spruce pedigree contains a large number of ungenotyped individuals (5,525 total; 1,218 genotyped), so the H matrix is needed to incorporate pedigree information for candidates lacking genotypes. In the Loblolly pine dataset, a much higher proportion of candidates were genotyped, making a G-matrix analysis sufficient for the main comparisons. We have added this explanation to the Methods.

For the H-matrix blending parameters, we used $\tau = 1$ and $\omega = 1$ (defaults in AGHmatrix, equivalent to Legarra et al. 2009). This parametrisation follows $H^{-1} = A^{-1} + [0 \;\; 0; 0 \;\; \tau G^{-1} - \omega A^{-1}_{22}]$, so $\tau = \omega = 1$ yields the standard Legarra–Misztal blending. This is now stated explicitly in the Methods.

> *Why was there a need to use a complex Bayesian mixture model (correlated continuous + categorical traits) for testing purposes? Is this really the standard model applied in the breeding programmes?*

Response:
The bivariate continuous-plus-categorical model was chosen for the QTL-MAS 2010 benchmark dataset because that dataset was designed with two correlated traits — one quantitative and one binary — and the workshop evaluation used both (Szydlowski et al. 2011). We agree that this combination is not the most common operational model; however, it serves our purpose of testing CVaR-OCS in a setting with explicit sex structure, a fully genotyped pedigree, and known true breeding values, all of which are essential for rigorous validation. In Norway spruce the model is a straightforward multivariate Gaussian GBLUP with three continuous traits, which is a standard approach in forest tree breeding. We have added a sentence to the Methods clarifying that the bivariate threshold model for QTL-MAS was chosen to match the original workshop design and that results on the quantitative trait are interpreted separately.

> *How do your genetic constraints of 0.02 and 0.03 transform into the standardly used constraint on rate of inbreeding per generation? And, how do the results affect or depend on the group coancestry constraint (Θ)?*

Response:
The group coancestry constraint Θ is related to effective population size N_e and inbreeding rate ΔF per generation by $\Delta F \approx 1/(2N_e)$. For the Norway spruce G-matrix analysis (n = 1,218), the average baseline coancestry among candidates is 0.014 (Table 1), so $\Theta = 0.02$ corresponds to approximately $\Delta F \approx 1\%$ per generation (target $N_e \approx 50$), within the range commonly recommended for forest tree breeding programs (typically $N_e \geq 50$).

Sensitivity to Θ is now directly addressed: the full CVaR-OCS efficiency frontier and solution metrics across $\Theta \in \{0.02, 0.03, 0.05\}$ for the QTL-MAS dataset are provided in Supplementary Table S5, showing consistent qualitative patterns (CVaR95 improvement of 0.6–3.4% at negligible gain cost at recommended operating points) across constraint levels.

> *When we entered the genomic era, there was a big question: which matrix should be used in the OCS step (A, G, H, ???), and which in the breeding value estimation step? Have you tested the sensitivity of your method?*

Response:
We have tested three relationship matrices (A, G, H) for Norway spruce in both the breeding value estimation step (via JWAS) and the OCS step. The overlap and agreement metrics across these combinations are reported in Supplementary Table S6. Using A vs. G in the OCS step yielded modestly worse MAP-OCS/MCMC-OCS agreement, while expanding to the full H-matrix population (n = 5,525) further reduced consistency (mean overlap 14.1 and 10.4 for H- and A-matrix analyses respectively), reflecting greater within-family ranking ambiguity at larger sample sizes.

We have added a paragraph to the Discussion acknowledging that the theoretical properties of OCS assume IBD-based kinship, but that genomic matrices are increasingly preferred in practice to capture within-family variation, and that the choice of matrix affects the interpretation of the coancestry constraint. This point was also raised by Reviewer 3 (see below).

> *I am worried about the extremely low number of MCMC samples. The authors use just 1000, whereas in real livestock datasets, the orders are in the 100,000s. There is also a lack of a proper assessment of the computational complexity of that method.*

Response:
We have substantially expanded the treatment of MCMC chain length and convergence diagnostics. A new Supplementary subsection (*MCMC chain length and convergence diagnostics*, Section S1.2) reports:

- For Norway spruce (G-matrix, n = 1,218): total chain length 52,000; 5,000 post-burn-in samples retained after thinning by 10. Effective sample sizes (ESS) for all heritability parameters exceeded 627 (ESS/n ≥ 0.125); trace plots confirmed stationarity (Figures S3–S4).
- For Norway spruce (H-matrix, n = 5,525): ESS was 216–226 for the two primary growth traits; ESS of 55–69 for the low-heritability Sprant17 and Lev17 traits, reflecting a flat likelihood surface (confirmed by trace plots, not non-stationarity). An extended chain (chain_length = 300,000) has been initiated.
- For QTL-MAS 2010: 5,000 post-burn-in samples (chain 52,000; burn-in 2,000; thinning 10).

We agree that I = 1,000 is modest and we have increased the length to 5000 iterations now as well as carefully assessing convergence. Note that the CVaR-OCS framework is in principle compatible with any I, and the Supplementary discussion notes that CVaR tail estimation precision scales with I.

Regarding computational scalability, Supplementary Table S5 (solver benchmarks) reports wall-clock times for all four solvers (COSMO, SCS, ECOS, Ipopt) across all matrix types and sizes. For the main CVaR-OCS analyses, the additional computational cost relative to MAP-OCS is O(I) linear constraints added to the QP: for I = 5,000 scenarios and n = 900 candidates, COSMO solves the CVaR-OCS problem in approximately 8–12 seconds on a standard workstation,

compared to <1 second for MAP-OCS. We have added a computational complexity paragraph to the Methods.

Minor Comments

Page 2, Line 35: The term "underestimate" may not always be appropriate.

Response:
Corrected. The sentence now reads: "[...] can lead to over- or underestimation of optimized contributions relative to the true optimum, depending on the direction of EBV error for the selected candidates."

Page 2, Lines 56–58: The statement that "ignoring uncertainty of EBV leads to suboptimal decisions" needs refinement. EBVs are unbiased estimators; the issue is ignoring uncertainty in the selection process.

Response:
We fully agree and have revised the sentence to: *"ignoring the uncertainty of EBVs in the selection process can lead to suboptimal decisions, even when the point estimates themselves are unbiased."*

Page 4, Line 83 and Page 5, Line 29: References to Section 2.6 and Section 2.5 appear incorrect. Please check internal cross-referencing throughout the manuscript.

Response:
All internal cross-references have been audited and corrected. Section numbers referenced in the text now correspond to the actual sections in the submitted document.

Figure 2c: It seems to be mentioned but not explicitly referenced in the text.

Response:
An explicit reference to Figure 2c has been added to the Results section (MCMC-OCS characterizes the impact of EBV uncertainty paragraph).

Table 4 (Overlap metric): It is unclear whether overlap is reported as a count or a percentage. Values such as 26.4 individuals are not meaningful. Similarly, 17.5 individuals are confusing.

Response:
The overlap metric is a mean count (expected number of individuals shared between MAP-OCS and MCMC-OCS top 100), averaged across 1,000 MCMC iterations, so non-integer values are appropriate and correctly interpreted as expectations. We have updated the table header and footnote to clarify: *"Mean overlap (±SD): expected number of individuals shared between MAP-OCS and MCMC-OCS top-100 selections, averaged over 1,000 MCMC iterations; non-integer values reflect iteration-level averaging."* We have also removed all percentage framings of this count to avoid confusion.

The classification of low, medium, and high overlap is unclear and somewhat subjective.

Response:
We have removed the low/medium/high overlap classification from the main text. Results are now reported as raw counts and Jaccard similarities, with comparisons made numerically rather than via qualitative labels.

The data availability statement includes only Norway spruce; please add a statement on other datasets and general code availability.

Response:
The Data Availability section has been expanded:

- Norway spruce phenotypic/genotypic data: available at https://github.com/jonhar97/MCMC_uncertainty_OCS/ (with recoded IDs for anonymisation).
- QTL-MAS 2010 dataset: publicly available from the original workshop repository (Szydlowski et al. 2011).
- Julia code for all analyses (CVaR-OCS, MCMC-OCS, robustness scores, figures): deposited at the same GitHub repository with a permanent DOI via Zenodo upon acceptance.

Reviewer 2

1. Equation 8 introduces a generic random effect a, but Equation 9 switches to g1 and g2 without explicitly linking them. The covariance notation in L25–26 and L37 is partly inconsistent; is the joint covariance Cov(y) dimensionally complete without the relationship matrix G? The residual term changes the notation from R to R0 which causes confusion. In L36, should the correlation formula be defined as covariance divided by square root of product of variances?

Response:
Thank you for the detailed notation audit. The notation has been fully unified throughout the Methods section:

- The mixed model (Eq. 8) now uses a for the generic random effect, and the subsequent bivariate model (Eq. 9) explicitly defines a1 and a2 as the vectors of breeding values for the continuous and categorical traits respectively, with a sentence bridging the two equations.
- The joint phenotypic covariance is now written in full: $Cov(y) = Z(G0 \otimes \Sigma)Z^{T} + R$, where R is the residual covariance matrix. R0 has been replaced by R throughout to avoid the dual-notation confusion.
- The genetic correlation formula now reads $rg = \sigma a1,a2 / \sqrt{(\sigma^2 a1 \cdot \sigma^2 a2)}$ in full.

2. The Bayesian setup is underreported. It is mentioned that the authors used JWAS with "default settings" and 1000 MCMC iterations, but they do not show the priors for fixed effects, variance components, etc.

Response:
We agree. The Methods now includes a dedicated paragraph on the JWAS prior structure: flat (improper) priors on fixed effects; scaled inverse chi-squared priors on variance components (v = 4.2, scale inferred from phenotypic variance following JWAS defaults; Cheng et al. 2018); and a Wishart prior on the genetic covariance matrix. MCMC chain settings are now reported in a consistent format for each dataset: burn-in, thinning, retained samples, and total chain length.

3. The Spearman correlation between cMAP and cj is less convincing. Many individuals may have close to zero contributions in OCS and rank correlation over individuals can be dominated by ties.

Response:
This is a valid concern. In the revised manuscript we have deprioritised the Spearman correlation between full contribution vectors (cMAP vs. c(j)) and instead emphasise the Spearman correlation between MAP EBV and MCMC-OCS selection frequency (reported as ρ = 0.782 for Norway spruce G-matrix), which is not subject to the tie-domination problem since EBVs vary continuously. We have added a note to the Results that the contribution-vector Spearman is reported for completeness but is susceptible to tie inflation; the overlap and Jaccard metrics are our primary agreement statistics.

4. The robustness score is actually a leave-one-out replaceability score since it is defined as the loss in expected gain when an individual is removed and OCS is rerun. The reason for a low score can be that a candidate has many close substitutes, not because of uncertain EBV.

Response:
This is an astute observation and we thank the reviewer for raising it. The robustness score as defined does indeed conflate two distinct sources of low scores: (a) high uncertainty in individual i's EBV across MCMC draws, and (b) the availability of close substitutes in the candidate pool. We have added a paragraph in the Discussion acknowledging this and reframing the interpretation:

> *"A low robustness score can reflect either high EBV uncertainty for that individual across MCMC iterations, or the presence of near-equivalent substitutes in the candidate pool — or both. The score therefore measures replaceability-under-uncertainty rather than EBV uncertainty alone. This distinction is practically relevant: an individual with a highly uncertain EBV but no close substitutes may still receive a high robustness score, while an individual with a stable EBV but several genetic clones may receive a low score. CVaR-OCS does not share this ambiguity, as it responds directly to the joint posterior uncertainty of the entire candidate pool."*

5. Low overlap between MAP-OCS and MCMC-OCS is very interesting to observe, but this does not automatically prove that MCMC-OCS gives better results. We may need simulation with known truth or external validations to prove this.

Response:
We fully agree, and this was also the primary concern of Reviewer 1 and the Associate Editor. The addition of the QTL-MAS 2010 simulation study directly addresses this. Moreover, we have revised the framing in the Abstract and Discussion to be explicit: MCMC-OCS is presented as a *diagnostic* tool (characterising instability of MAP-OCS) rather than a method that is itself superior to MAP-OCS. CVaR-OCS is the recommended decision-making framework, and its superiority is validated against the oracle MAP-OCS(TBV) solution in the simulation study. The Discussion concludes that multi-generation simulation studies are still needed to characterise long-term effects.

Reviewer 3

> *1. The robustness score threshold is arbitrary and underexplored. The authors explicitly acknowledge that the bottom 25% cutoff is arbitrary. However, I would add some more intuition/explanation to guide a practical decision by someone implementing this tool in the discussion section.*

Response:
Thank you. We have added guidance in the Discussion on how a practitioner might choose the exclusion threshold:

> *"The bottom-quartile threshold used here is illustrative. In practice, the threshold could be guided by: (i) a tolerable budget of genetic gain loss (e.g., exclude individuals until further exclusion would cost more than 1% gain); (ii) the structure of the robustness score distribution, which often shows a natural gap between a small group of markedly low-scoring individuals and the remaining candidates; or (iii) the efficiency frontier of CVaR-OCS itself, which provides a principled and continuous parameterisation of the robustness–gain trade-off through α and μ. We recommend CVaR-OCS as the primary decision-making tool, with robustness scores serving as supplementary diagnostics rather than hard decision rules."*

> *2. The paper uses G (IBS-based) and A (IBD-based) matrices somewhat interchangeably in the OCS constraint, but the theoretical properties of OCS assume IBD-based kinship. The authors show that using A vs. G changes overlap metrics, but they do not discuss what this means for the interpretation, given that the same threshold was used for coancestry.*

Response:
This is an important theoretical point. We have added a paragraph to the Discussion:

> *"The group coancestry constraint in OCS was originally derived under the assumption of IBD-based kinship. Using IBS-based genomic relationship matrices G instead of pedigree-based A changes the parametric space of the constraint: G_{ij} measures allele sharing across the genome (including a baseline level reflecting founder allele frequencies), whereas A_{ij} measures the probability of IBD relative to a pedigree base. Applying the same Θ to both matrices therefore implies different effective inbreeding targets. In Norway spruce, the baseline average coancestry was 0.014 (pedigree) vs. 0.012 (genomic), so the numerical effect is modest at Θ = 0.02 but would become more*

> *pronounced at tighter constraints or in more inbred populations. Researchers wishing to ensure comparability between G- and A-based OCS should either calibrate Θ to match the effective inbreeding target, or use the H matrix, which inherits the IBD interpretation from A."*

> *3. The increase in Gini coefficient after excluding high-risk individuals is mechanistically explicable but exposes a conceptual tension in the dual-metrics framework: individual robustness and portfolio concentration do not necessarily move together, and the paper provides no principled guidance on how to weight these competing dimensions.*

Response:
We agree and have substantially revised the Discussion paragraph on dual metrics to address this tension directly. The revised passage explains the mechanism: removing candidates forces the constrained OCS to concentrate contributions among the remaining high-EBV individuals, reintroducing fragility elsewhere in the portfolio. Crucially, we now present this as the motivating argument for CVaR-OCS: unlike post-hoc exclusion, CVaR-OCS simultaneously broadens the selection base, reduces concentration, and improves tail-gain security. The contrasting behaviour between Norway spruce (divergent metrics) and QTL-MAS (concordant) is noted and attributed to the tighter coancestry constraint and smaller candidate pool in Norway spruce. We acknowledge that which metric should take precedence in a specific program depends on operational priorities (diversity vs. expected gain), and that further multi-generation simulation work is needed to determine the long-term consequences of each strategy.

> *4. Figure 3b shows some high-contribution individuals flagged as high-risk; this is one compelling result and deserves more discussion.*

Response:
We agree this is one of the most practically important findings. The revised Results section now includes an explicit discussion:

> *"Notably, three of the top-25 MAP contributors were classified as high-risk, including one in the top-10 — illustrating that high contribution under MAP EBVs does not guarantee stability under posterior uncertainty. An individual can receive a large MAP contribution because their point-estimate EBV is high relative to the coancestry constraint, while their full posterior distribution overlaps substantially with other candidates, making them replaceable in many MCMC iterations. This is precisely the class of selections that CVaR-OCS is designed to penalise."*

Figure 3b (robustness score vs. MAP contribution) is now referenced explicitly in this context.

> *5. Page 5, line 80: It seems that the constraint mentioned here is in relation to the Loblolly pine dataset. However, the authors already mentioned this in lines 62–63 (Page 3).*

Response:
The duplicated constraint statement has been removed from Page 5. The constraint is now stated once, in the appropriate case-study subsection.

> *6. Page 13, line 67: The authors claimed a "practical decision-making framework" based on the 25% threshold, which was arbitrary. Tune down this sentence.*

Response:
The sentence has been revised to: *"We have also provided illustrative robustness metrics and replacement strategies that may assist breeders in identifying high-risk selections under EBV uncertainty; the principled CVaR-OCS framework provides the recommended approach for operational programs where a formal uncertainty-aware objective is desired."*

7. Page 4, line 83: (Section 2.6) could not find this section.

Response:
The erroneous cross-reference has been corrected (see also Reviewer 1 minor comment on cross-references).

Summary of Manuscript Changes

1. New simulation study: QTL-MAS 2010 analysis with n = 900 candidates, 5,000 MCMC samples, known TBVs, and oracle comparison (new Results subsection, Table 2, Figure 5).
2. CVaR-OCS reframing: the primary contribution is now presented as CVaR-OCS; MCMC-OCS and robustness scores play a supporting diagnostic role.
3. Ad-hoc exclusion benchmark: post-hoc robustness-based exclusion is directly compared to CVaR-OCS in Norway spruce (Table 3, last row), demonstrating the superiority of the principled approach.
4. Notation unified: a vs. g, R vs. R0, capital C renamed to marginal gain M.
5. MCMC diagnostics expanded: new Supplementary Section S1.2 with ESS, trace plots, and chain-length justification for all datasets.
6. Dataset description: new Table 1 and Supplementary Table S1 with full phenotypic data summary.
7. Cross-references corrected throughout the manuscript.
8. Discussion expanded: A/G/H matrix interpretation, robustness score replaceability interpretation, dual-metric tension, high-risk high-contributor finding, threshold guidance.
9. Data availability expanded: QTL-MAS data and code repository added.
10. References corrected: Fogg et al. arXiv ID and Pocrnic et al. book title added.

June 30, 2026
RE: GENETICS-2026-309564

Dear Dr. Ahlinder:

I am pleased to accept your manuscript titled "Uncertainty-aware breeding decisions: MCMC-based optimum contribution selection increases breeding decision robustness" for publication in GENETICS, pending minor revision and fulfillment of the data availability requirements.

Thank you for the thorough response to the reviewer comments. The new methodology CVaR-OCS is innovative in the genetics research community, and the expanded Discussion addresses several important issues.

Please submit your revision along with a brief description of how you modified the manuscript in response to the reviewers' concerns and suggestions (which can be viewed at the bottom of this email). No additional analyses are required, nor do I see the need for a separate Conclusions, but I would like to see how you address the other comments of Reviewer #1.

I expect you should be able to submit a revised manuscript within 30 days. A suitably revised manuscript will be acceptable for publication; I don't expect to send it out for review.

Prior to uploading your revised manuscript please format it according to GENETICS style and ensure you have all required elements. Author Guidelines are at https://academic.oup.com/genetics/pages/author-guidelines#section-12. These guidelines have been updated with new requirements and your careful attention is required to avoid delays at the source file stage post-acceptance.

Follow this link to submit the revised manuscript: Link Not Available

Thank you for submitting this story to Genetics.

Sincerely,

Jeffrey Endelman
Associate Editor
GENETICS

Approved by:
Hongyu Zhao
Senior Editor
GENETICS

Reviewer comments:
Reviewer #1 :

As I mentioned in round 1, method is worthwhile and timely, and the paper makes a useful contribution to the growing literature on uncertainty-aware breeding decisions. My main suggestions concern the positioning of CVaR-OCS relative to existing Robust OCS approaches, and the precision of several comparative claims.

Regarding the rebuttal, the authors have addressed the reviewers' comments comprehensively, but have also significantly changed the manuscript (as can be seen in the track changes version), which did not necessarily improve it and unfortunately raised several new questions.

Major comments:

What is the justification for dropping one dataset completely that was present in the initial version? The Loblolly pine is not any more assessed, which is strange, given the comments to authors include the responses to it. Why remove it?

Benchmarking is incomplete, as the manuscript positions CVaR-OCS mainly against point-estimate OCS and an ad hoc exclusion strategy. However, the most relevant comparator is the recent robust OCS of Fogg et al. (2024). Since the paper explicitly argues that CVaR-OCS improves on that framework by using the full joint posterior, this should be shown empirically rather than discussed only conceptually.

More generally, I think the comparison between CVaR-OCS and Robust OCS would benefit from a clearer conceptual discussion. The key distinction is not simply whether uncertainty is included, but how uncertainty is represented and how conservation is controlled in the optimization. Robust OCS and CVaR-OCS differ in their uncertainty representation, in how the uncertainty enters the optimization, and in how risk aversion is tuned. In my reading, Robust OCS can be more conservative than CVaR-based optimization, depending on the uncertainty specification and tuning parameter, while some robust formulations may also be more scalable than scenario-based CVaR. Clarifying these trade-offs would help readers understand when each approach may be preferable.

p. 2, lines 33-38
The statement that Robust OCS characterizes EBV uncertainty only marginally seems too broad. That description may apply to an SE-based or diagonal uncertainty specification, but robust OCS can also be built using covariance information (prediction error covariances), in which case uncertainty is not treated independently across candidates. Please clarify whether the comparison is intended specifically for the SE-based/diagonal implementation rather than for Robust OCS more generally.

p. 2, lines 71-80
The manuscript emphasizes computational practicality mainly relative to per-scenario approaches such as Pocrnic et al. (2023) and CVaR-OCS. It would strengthen the paper to also discuss scalability relative to other uncertainty-aware optimization approaches, especially covariance-based Robust OCS formulations. Scenario-based CVaR grows with the number of posterior draws, whereas covariance-based robust formulations may have different computational scaling. A short discussion of this point would help place the proposed method more clearly within the broader literature.

p. 15, lines 38-48
The claim that CVaR-OCS "subsumes" Robust OCS appears too strong unless this is formally demonstrated or empirically evaluated. As the confidence level approaches one, CVaR approaches a worst-tail criterion over the empirical scenario set, but this is not necessarily equivalent to Robust OCS over a general uncertainty set Ω with its own risk-control parameter. Likewise, the statement that CVaR-OCS captures joint uncertainty whereas Robust OCS treats uncertainty independently is only valid for diagonal or SE-based uncertainty specifications, not necessarily for covariance-based robust formulations. Please either soften this claim or provide a clearer formal justification.

Choice of α and μ needs stronger justification. The elbow criterion is convenient, but it is still somewhat ad hoc. The authors should explain more clearly how this choice maps onto a breeder's real decision problem and acceptable trade-off between expected gain and robustness.

Am I correct that the MCMC-OCS results from simulation study are completely missing?

Given all above, I would argue you should add at least add a paragraph on limitations of the approach. You do cover computational issues I mentioned in the round 1, so thank you for that.

I may have overlooked it, but I did not find a dedicated Conclusions section. Even if the journal does not require a separate section, a brief concluding section or final summary paragraph would improve readability and reinforce the main take-home messages.

Minor:

The are bunch of inconsistencies, in Table 2 and rest of the text;
gain overestimation is reported as 16.7% in some places and 18.8% in Table 2
simulated-data recommended operating point is stated as α=0.95,μ=1.50, in the text, but Table 2 gives $ \alpha^=0.90, \mu^=1.8 $
Data statement - add link to simulation code for reproducibility
What is the context of estimating h^2 and for simulation study? You know true values. Does that somehow enters the equation?

Reviewer #3 :

They have addressed all of my comments.

Associate Editor comments:

Figure 1 - There is a disconnect between what I see in Figure 1 and how the results are described in the main text and caption. The description is very similar to the Norway spruce results in Figure 4 and makes sense there. But the frontier curves in Figure 1a do not have open diamonds, are not concave, and do not appear to be expressed in percentages.

I could not find the anonymized Norway spruce dataset on the GitHub page. This is a requirement for publication. I am glad you will also make the code available.

Response to Reviewers

GENETICS-2026-309564

We thank the Associate Editor and reviewers for their continued engagement with this manuscript and for the constructive comments that have substantially improved it. We address each point below.

Associate Editor comments

Comment: *Figure 1 does not match its caption — the frontier curves lack open diamonds, are not concave, and are not expressed as percentages.*

Response: Thank you very much for spotting this error and we have corrected this. Figure 1a now correctly displays genetic gain loss and $CVaR_{95}$ improvement as percentages relative to the MAP-OCS baseline, with open diamonds marking the elbow point on each α curve, identified via the geometric perpendicular-distance criterion described in Materials and Methods. This process brought to light an unrelated computational issue in how the MAP-OCS baseline gain was recorded in the underlying frontier output (a posterior-mean-scale objective value had been used rather than the correct in-sample scenario-mean value); this has been corrected at the source, and all reported frontier statistics for the QTL-MAS dataset have been independently re-verified against the corrected output using the final l = 5,000 MCMC chain.

Comment: *The anonymized Norway spruce dataset was not found on the GitHub repository.*

Response: The anonymized Norway spruce dataset (both the n = 1,218 genotyped subset and the n = 5,525 full pedigree), and all associated analysis scripts are in place in the GitHub repository with instructions how to run the analyses.

Reviewer #1 — Major comments

Comment: *Why was the Loblolly pine dataset dropped from the previous revision?*

Response: We have restored the Loblolly pine case study, with full dataset description, CVaR-OCS results, and figures now presented in the Supplementary Material, with the main text noting the dataset and pointing readers to the supplement (Materials and Methods and Results). The Loblolly pine results are consistent with the Norway spruce and QTL-MAS findings, providing tail-gain protection at negligible expected-gain cost in a second, independent real-data breeding population.

Comment: *The comparison to Fogg et al. (2024) robust OCS should be empirical rather than only conceptual.*

Response: We agree this would be a valuable comparison. However, as no additional analyses were requested for this revision, and given the substantial empirical content already presented across three datasets, we consider a rigorous empirical comparison, requiring reimplementation of the covariance-based robust OCS formulation of Fogg et al. under matched conditions, better suited to a dedicated follow-up study. We have expanded our conceptual discussion (new Limitations paragraph, Discussion) to more precisely characterize where the two approaches are expected to diverge, including computational scaling and how each controls risk aversion.

Comment: *The conceptual distinction between Robust OCS and CVaR-OCS needs clarification, particularly regarding whether "marginal" uncertainty characterization applies to Robust OCS generally or only to SE-based/diagonal implementations.*

Response: We have revised the relevant text (Introduction) to specify that the marginal/independent uncertainty characterization applies to SE-based or diagonal implementations, and explicitly note that covariance-based Robust OCS formulations, including that of Fogg et al. (2024), which uses an ellipsoidal uncertainty set over the full posterior covariance, can encode correlated prediction errors. The distinction we draw is with implementations that treat uncertainty independently across candidates, not with Robust OCS as a class of methods.

Comment: *The claim that CVaR-OCS "subsumes" Robust OCS (p. 15) is too strong without formal or empirical justification.*

Response: We agree and we have softened this claim throughout and added an explicit statement in the new Limitations paragraph noting that a formal or empirical comparison of the two paradigms, including how each

controls risk aversion (CVaR confidence level and penalty weight vs. uncertainty-set radius), is an important direction for future work, rather than asserting subsumption.

Comment: *Computational scalability should be discussed relative to covariance-based Robust OCS formulations, not only per-scenario approaches.*

Response: Addressed in the new Limitations paragraph (Discussion), which notes that scenario-based CVaR-OCS scales with the number of posterior draws, whereas covariance-based robust formulations summarize uncertainty through a single (co)variance structure and may scale more favourably for very large candidate sets.

Comment: *Choice of α and μ via the elbow criterion needs stronger justification relative to a breeder's actual decision problem.*

Response: We have added an explicit acknowledgment in the new Limitations paragraph in the Discussion that the elbow criterion, while practical and reproducible, is a heuristic that does not directly encode a breeder's utility function, and noted that linking these parameters to economic weights (Hazel 1943; Simianer et al. 2023) or to formal elicitation of risk preferences (cf. Akdemir and Sánchez 2016) would provide a more principled operating point.

Comment: *Are the MCMC-OCS results from the simulation study completely missing?*

Response: We have added a standalone MCMC-OCS diagnostic for the QTL-MAS dataset, positioned in the Results immediately before the CVaR-OCS parameterisation section, consistent with the paper's stated logic that this diagnostic motivates CVaR-OCS. MAP-OCS was independently re-solved against each of the l = 5,000 posterior EBV scenarios; the resulting solutions showed a mean overlap of 13.4 individuals with the MAP-OCS(EBV) reference solution (mean Jaccard similarity 27.4%), and a strong rank correlation between EBV and selection frequency (Spearman ρ = 0.875), with 229 of 900 candidates (25.4%) never selected across the 5,000 independent re-optimisations. This substantial per-scenario instability directly motivates the CVaR-OCS approach and closes the gap the reviewer identified.

Comment: *A limitations paragraph should be added.*

Response: Added (Discussion), covering computational scaling of scenario-based CVaR relative to covariance-based robust formulations, the scope of the "marginal uncertainty" comparison, the heuristic nature of the elbow criterion for selecting α and μ, and the single-generation scope of the oracle validation.

Comment: *A brief concluding section or paragraph would improve readability.*

Response: Added as the final paragraph of the Discussion, consistent with the Associate Editor's note that a separate Conclusions section is not required.

Reviewer #1 — Minor comments

Comment: *Inconsistent gain overestimation figures (16.7% vs. 18.8%) and inconsistent recommended operating point (α = 0.95, μ = 1.50 in text vs. α* = 0.90, μ* = 1.8 in Table 2).*

Response: On investigation, both the 18.8% figure and the α/μ mismatch traced to a single underlying issue: the reported "overestimation" compared MAP-OCS's optimization objective (computed on the posterior-mean scale) against an in-sample scenario-mean evaluation on a different standardization scale, which is a methodological artifact rather than a substantive finding. We have corrected this at the source and reframed the discussion of MAP-OCS's optimism around the oracle comparison, where MAP-OCS(EBV) realises 78% of the oracle-achievable gain at Θ = 0.03, rather than the scale-inconsistent in-sample figure. In direct response to this reviewer's concern (raised in the previous round) that MCMC chains of only a few hundred to a thousand iterations are inadequate, we have also re-run the QTL-MAS analysis using the full l = 5,000 post-burn-in posterior (52,000 total iterations, 2,000 burn-in, thinned to 5,000), matching the chain length already used for Norway spruce and Loblolly pine; effective sample sizes for all heritability and genetic (co)variance parameters exceeded 200 (Supplementary Material). The recommended operating point at Θ = 0.03 is now α = 0.95, μ = 1.25, which achieves the highest CVaR95 improvement (+0.68%) at negligible gain cost (−0.12% in-sample). All numeric values in Table 2, Table S7, Figure 1, and the corresponding results text have been rebuilt from this corrected, full-length chain and independently re-verified against the underlying solver output. We further note in the Discussion that the CVaR95 improvement is smaller and more precisely estimated with the longer chain than with the shorter one used previously, consistent with the disproportionate sensitivity of tail-risk metrics to the number of posterior draws informing the tail.

Comment: *The data availability statement should include a link to the simulation code.*

Response: The reproducibility repository, including simulation code for all three datasets, is being finalized and will be in place prior to submission.

[PENDING — GitHub repository upload — same status as the Associate Editor's comment above]

Comment: *What is the context of estimating h^2 for the simulation study, given the true values are known? Does this enter the analysis?*

Response: Heritability and genetic correlation were estimated from phenotypic and genomic data via the Bayesian GBLUP model, with no use of simulated true breeding values in model fitting; TBVs entered the analysis only in the subsequent oracle validation. We have extended the relevant passage to explicitly compare our estimates against the true simulated values reported by Szydłowski and Paczyńska (2011): the posterior-mean heritabilities (0.46 for the quantitative trait, 0.47 for the binary trait, fitted as a threshold model) and genetic correlation (0.69) closely recovered the true simulated values (h^2 = 0.39–0.52, sex-specific, and 0.48, respectively; genetic correlation 0.59–0.68), confirming that the model correctly recovered the underlying genetic architecture.

Reviewer #3

We thank the reviewer for their positive assessment and confirmation that all previous comments have been addressed.

July 19, 2026
RE: GENETICS-2026-309564R1

Dr. Patrik Waldmann
Oulun yliopisto
Mathematical Sciences Research Unit
P. O. Box 8000
Oulu, N/A 90014
Finland

Dear Dr. Waldmann:

Congratulations, your manuscript titled "Uncertainty-aware breeding decisions: MCMC-based optimum contribution selection increases breeding decision robustness" is accepted for publication in GENETICS! Many thanks for submitting your research to the journal.

It was kind of you to acknowledge the comments of the editor and reviewers. Please note the correct spelling of Endelman (not Endelmann).

To Proceed to Publication:
1. Format your article according to GENETICS style: https://academic.oup.com/genetics/pages/author-guidelines

2. Ensure that you comply with data and community resource citation guidelines:
https://academic.oup.com/genetics/pages/author-guidelines#section-5-9-2

3. Upload your final files at https://genetics.msubmit.net

4. Add oupsupport@scipris.com and genetics.oup@novatechset.com (or the domains @scipris.com and @novatechset.com) to your email program's "safe senders" list. You will be contacted by both at various points during the production process.

Notes:
- Your currently-accepted manuscript (unedited, as submitted, reviewed, and accepted) will be published at GENETICS and deposited into PubMed as an Advance Access article. Notify sourcefiles@thegsajournals.org before signing your license if you do not wish to publish your article via Advance Access.

- We invite you to submit an original color figure related to your paper for consideration as cover art. Please note that submissions created with AI image generation tools are not eligible. Please email your submission to the editorial office or upload it with your final files. You can submit a small-sized image for evaluation, and if selected, the final image must be a TIFF file 2513px wide by 3263px high (8.375 by 10.875 inches; resolution of 600ppi). Please avoid graphs and small type.

- After files are sent to Oxford University Press we use SciPris to manage article licensing and payment. If you do not have a SciPris account, you will receive an email from no-reply@scipris.com to sign up to use Oxford University Press' author portal. After logging in, follow the online instructions to sign your license and arrange any payment due.

If you have any questions or encounter any problems while uploading your accepted manuscript files, please email the editorial office at sourcefiles@thegsajournals.org.

Sincerely,

Jeffrey Endelman
Associate Editor
GENETICS

Approved by:
Hongyu Zhao
Senior Editor
GENETICS
